# Surface-based analysis increases the specificity of cortical activation patterns and connectivity results

**DOI:** 10.1038/s41598-020-62832-z

**Published:** 2020-03-31

**Authors:** Stefan Brodoehl, Christian Gaser, Robert Dahnke, Otto W. Witte, Carsten M. Klingner

**Affiliations:** 1 Hans Berger Department of Neurology, Friedrich Schiller University Jena, Jena, Germany; 2 Biomagnetic Center, Friedrich Schiller University Jena, Jena, Germany; 3 Department of Psychiatry, Friedrich Schiller University Jena, Jena, Germany

**Keywords:** Neuroscience, Computational neuroscience, Cortex

## Abstract

Spatial smoothing of functional magnetic resonance imaging (fMRI) data can be performed on volumetric images and on the extracted surface of the brain. Smoothing on the unfolded cortex should theoretically improve the ability to separate signals between brain areas that are near together in the folded cortex but are more distant in the unfolded cortex. However, surface-based method approaches (SBA) are currently not utilized as standard procedure in the preprocessing of neuroimaging data. Recent improvements in the quality of cortical surface modeling and improvements in its usability nevertheless advocate this method. In the current study, we evaluated the benefits of an up-to-date surface-based smoothing in comparison to volume-based smoothing. We focused on the effect of signal contamination between different functional systems using the primary motor and primary somatosensory cortex as an example. We were particularly interested in how this signal contamination influences the results of activity and connectivity analyses for these brain regions. We addressed this question by performing fMRI on 19 subjects during a tactile stimulation paradigm and by using simulated BOLD responses. We demonstrated that volume-based smoothing causes contamination of the primary motor cortex by somatosensory cortical responses, leading to false positive motor activation. These false positive motor activations were not found by using surface-based smoothing for reasonable kernel sizes. Accordingly, volume-based smoothing caused an exaggeration of connectivity estimates between these regions. In conclusion, this study showed that surface-based smoothing decreases signal contamination considerably between neighboring functional brain regions and improves the validity of activity and connectivity results.

## Introduction

**Figure 1:**
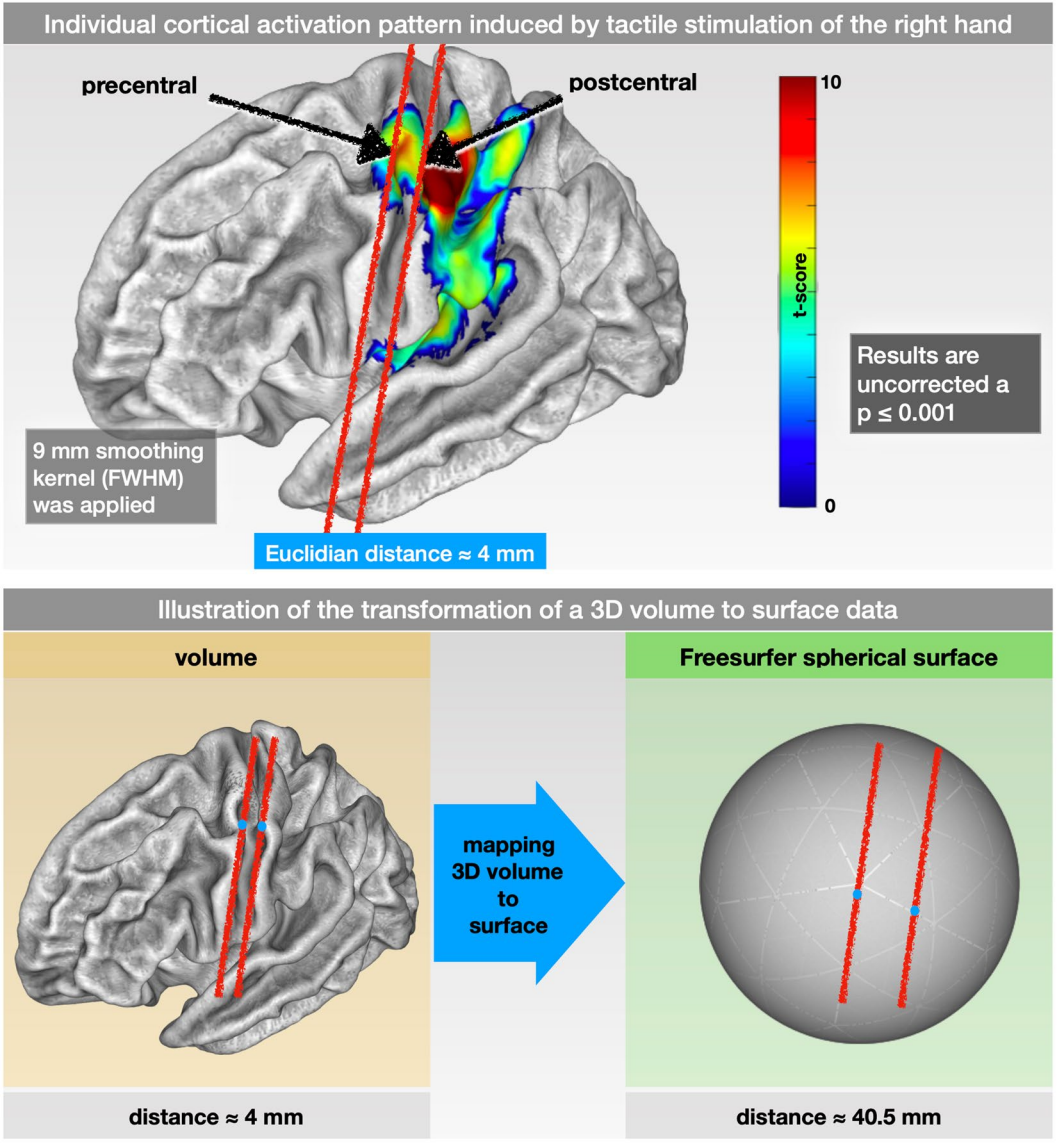
Individual cortical activation pattern (SPM maps) due to tactile stimulation and illustration of the transformation of a 3D volume to surface data. (Upper row) Individual cortical activation pattern (SPM maps) induced by tactile stimulation of the fingers of the right hand using a 9 mm FWHM (full width at half maximum) Gaussian smoothing kernel. The arrows point to 2 exemplary locations within the primary motor and the primary somatosensory cortex. However, being located in 2 different functional (motor vs. sensor) and anatomical (pre- vs. postcentral gyrus) structures, their Euclidean distance within the 3D space was only approximately 4 mm. (Lower) Illustration of the transformation of a 3D volume to surface data. The 2 exemplary locations within the pre- and postcentral gyrus in the original 3D volume are approximately 4 mm apart. By mapping the 3D volume to the surface space (FreeSurfer spherical template), the same 2 points were approximately 40 mm apart.

Over the past decades, functional brain imaging has become the leading tool to localize brain functions and to decode the interactive relationship between specific brain areas. Both tasks require data analyses that assume we can distinguish the signal time course between different brain areas. However, a common problem is the contamination of the signal time course of one area with that of another. This remains a major challenge, particularly in the analysis of MEG and EEG data where this problem has received much attention^1^. Although functional magnetic resonance imaging (fMRI) data are considered to be less affected by this problem, spatial smoothing can induce the same signal contamination in brain areas that are close together. Spatial smoothing is a standard preprocessing step in fMRI data analyses. It is usually performed using volumetric smoothing by a three-dimensional Gaussian filter with a full width at half maximum (FWHM) size of several millimeters, denoted as “kernel” size^2^. Clearly, smoothing leads to a mixture of the signal time courses of voxels captured by this filter. This signal averaging can be beneficial if the voxels belong to the same functional region and increase the signal-to-noise ratio for information about larger-scale brain areas. However, due to the folding of the brain, the spatial 3D distance from one functional brain area to another could be small. Figure 1 shows an example of two localizations within the pre- and postcentral gyrus that were only 4 mm apart but belonged to different functional systems (primary motor and primary somatosensory cortex). Therefore, even small spatial filters with kernels of 3 to 9 mm might cause contamination of somatosensory brain areas by motor signals and vice versa. Studies have estimated a smoothing kernel size of ~8 mm to optimize cortical activation strength in robust experiments while even greater smoothing kernels are suggested for less robust experiments^3^. The chosen smoothing kernel did not only affect the size and strength of the modeled brain activity but also causes changes in the localization of functional activations^4^.

The problem of a mixture of signals induced by smoothing is also important for analyses of the connectedness between brain areas. Currently, measures of functional connectivity estimated by correlation analyses are the most popular approach, and it is particularly sensitive to any mixture of signals. Therefore, volume-based spatial smoothing can severely affect the results of connectivity analyses of neighboring brain regions.

A proposed improvement is the use of two-dimensional smoothing on the unfolded cortex (see Fig. 1 for an illustration). This might be particularly helpful for the analysis of brain areas that are close together in the folded brain but are more distant in the unfolded brain, such as the primary motor and primary somatosensory cortex. Moreover, the restriction of smoothing to the cortical surface might provide more sensitive results for cortical activations due to the exclusion of white matter and cerebrospinal fluid. The method of surface-based smoothing has been proposed previously^5–8^ but is currently not utilized as a standard procedure in the preprocessing of neuroimaging data. Although there are standardized pipelines (i.e., FreeSurfer fs-fast stream or Human Connectome Project) to perform functional fMRI analysis on the cortical surface, this method is currently not utilized as a standard procedure in the preprocessing of neuroimaging data^8,9^. Reasons for the reluctance to use SBAs (SBA) might be problems with its usability and variations in the quality of the underlying surface modeling. However, the modeling of the cortical surface, as well as its usability, has evolved and improved in recent years^10,11^. Recent approaches have even employed resolutions of <1 mm and combined T1 and T2 images for surface generation^8^.

Here, we compared the effects of volume-based and surface-based smoothing using state of the art cortex surface modeling derived from the Computational Anatomy Toolbox (CAT12; http://dbm.neuro.uni-jena.de/cat/). We were particularly interested in the contaminant effects of pure somatosensory stimulation on the primary motor cortex and its implications for the study of brain activity and connectivity. For this aim, we employed fMRI during a tactile stimulation paradigm in 19 subjects. Furthermore, we used simulated data to more comprehensively understand the effects of spatial smoothing in comparison to real world data.

## Methods

### Subjects

Nineteen subjects (10 male, age 27.5 ± 6.7 years) participated in the current study. All participants were right-handed and free of any neurological diseases. The study was approved by the local ethics committee (FSU Jena / reference number 4301-01/15), and all subjects gave their written informed consent according to the Declaration of Helsinki.

### MRI acquisition

All experiments were performed on a 3.0-T MR scanner (Trio, Siemens, Erlangen, Germany) to obtain echo-planar T2*-weighted image volumes (EPI) and trans-axial T1-weighted structural images. The EPI images (voxel size = 3 mm × 3 mm × 3 mm; repetition time = 2.52 s; TE = 35 ms; 40 trans-axial slices) covered the entire cerebrum and cerebellum. The high-resolution T1-weighted structural images had a voxel size of 1 mm × 1 mm × 1 mm to allow for precise anatomical localization.

### Principal data analysis

Data analysis was performed on a Windows 7 PC using MATLAB R2016a (MathWorks, Natick, MA) and Statistical Parametric Mapping (Version 12, Wellcome Department of Cognitive Neurology, London, UK, http://www.fil.ion.ucl.ac.uk/spm). For normalization, segmentation and surface mapping, the CAT12 toolbox of the Structural Brain Mapping Group (http://www.neuro.uni-jena.de/cat/) was employed^12^.

### General linear model of task-induced and simulated local brain activity

#### Analysis strategies

**Figure 2:**
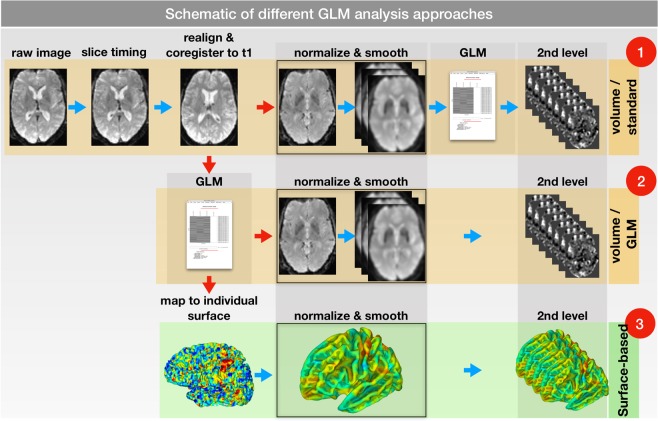
Schematic of different GLM analysis approaches. All approaches started from the same fMRI data. Consequently, a slice time correction, a realignment and a co-registration to the corresponding structural T1 image were performed. (1) In the first approach, the functional images were normalized and smoothed (to 6, 9 and 12 mm) before applying the GLM. (2) In the second approach, the GLM was performed before normalization and smoothing. (3) In the third approach, the GLM was performed in the individual space (as in approach 2). Afterwards, the results were mapped to the individual surface and normalized to a standard surface. The smoothing occurred on the normalized surface. Group analyses for all 3 approaches were performed using a 1 sample t-test.

To compare volume and surface-based imaging analyses, we used a total of 3 different approaches, as shown in Fig. 2.

All approaches started with the rejection of the first 3 images, a slice timing, realignment^13^ and co-registration to the corresponding structural T1 image. To compensate for the lack of a proper fieldmap, we used a nonlinear registration between the structural and functional images as provided by the CAT12 toolbox. Furthermore, the corresponding T1 image was segmented and spatially normalized using a DARTEL template within CAT12 (surface mapping and deformations were stored for future use).

1. Volume-based (Fig. 2, row 1): In the standard volume-based approach (VBA), all fMRI time series images were normalized using the transformation matrix of the normalized T1 image. Afterwards, the normalized images were smoothed using different FWHM sizes of 6, 9 and 12 mm. The SPM model according to our tactile stimulation was applied on the smoothed images. Consequently, group analysis was performed using a 1 sample t-test.
2. Volume-based/general linear model (GLM) first (Fig. 2, row 2): To employ a VBA that is equal to the following SBA, within this approach, a SPM-based GLM analysis was performed before the normalization and the smoothing step. The results of the first level analysis were normalized using the same transformations as in the step above. Again, the normalized results were smoothed using FWHM sizes of 6, 9 and 12 mm. Consequently, the group analysis was performed using a 1 sample t-test.
3. Surface-based (SBA)(Fig. 2, row 3): Here, the SPM-based GLM analysis was performed upon the realigned functional images. The contrast files (con_00*.nii) of the first-level analysis were then mapped to their individual surface spaces using the previously generated surfaces of the individual T1 images. For the surface mapping of the fMRI data, we applied the absolute maximum option as recommended in the CAT12 toolbox.

Afterwards, the individual surfaces were mapped to a normalized template surface and smoothed with different FWHM sizes of 0, 3, 6, 9 and 12 mm using the CAT12 toolbox. A more detailed description of the surface registration procedures is provided by Yotter *et al*.^11^; as a standard surface, we used the 32k HCP-compatible mesh as provided in the CAT12 toolbox. The final group analysis was performed upon the normalized and smoothed surfaces using a 1 sample t-test.

#### Task-induced brain activity

Functional data were acquired while the participants (N = 19) were stimulated at fingers 2 and 4 of the right hand using a pneumatic driven device (airpuff). Stimuli lasted 1.5 seconds and were applied in a pseudo-randomized order (inter-stimuli interval 15–25 seconds, total of 40 stimuli and approximately 17 minutes). A total of 410 images was recorded.

#### Simulated HRF signal in the primary somatosensory cortex

**Figure 3:**
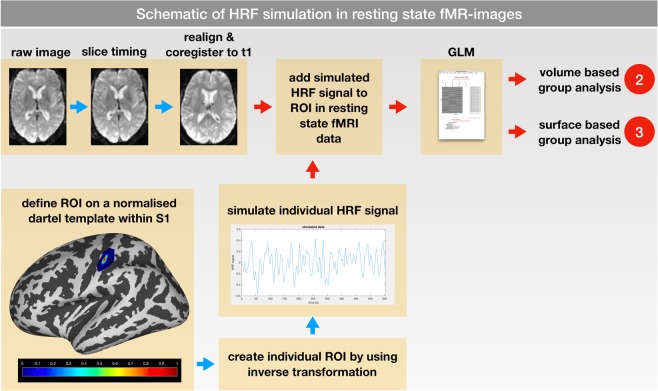
Schematic of HRF simulation. rs-fMRI data were co-registered to their corresponding structural t1 image. Using a DARTEL template in normalized space, a region of interest (ROI) was defined within the primary somatosensory cortex (S1); values of the ROI ranged from 1 (center) to 0 (6 mm distance) to modify the amplitude of the simulated HRF. For each subject an individual HRF signal was created using the simTB-Toolbox. After mapping the ROI to the individual space, the simulated HRF signals were added to individual rs-fMRI time series. Afterwards, the GLM analysis was performed within the individual space; consequently, the volume (2) and surface-based (3) group analyses were carried out.

Resting-state fMRI (rs-fMRI) of all 19 participants was employed (200 images each, TR 2.52 s). Using the fMRI Simulation Toolbox (simTB-Toolbox, available on http://mialab.mrn.org/software), we created individual hemodynamic response function (HRF) signals for each subject (signals occurred every 10 s and lasted 1 s) within the primary somatosensory cortex of each individual fMRI time series^14^. For a detailed description, please view Fig. 3.

Analogously, in a second simulation, we added two different signals to the same rs-fMRIs within the primary somatosensory cortex. According to the study of Martuzzi and colleagues^15^, we used the MNI coordinates of the centers of the cortical representation of fingers D1 (−48.4 × −19.0 × 52.7) and D3 (−44.2 × −21.3 × 56). Individual simulated HRF signals occurred in a randomized alternating order (every 10 s for 1 s).

Afterwards, the volume-based and surface-based GLM analyses were performed.

#### Multiple comparison correction

To overcome the problem of multiple comparisons, for all results from the second-level analysis, we calculated the threshold-free cluster enhancement (TFCE) and adjusted it to a threshold of p ≤ 0.01 familywise error (FWE) as provided in the CAT12 toolbox^12,16^.

For comparison, the results of the VBAs were consequently mapped to the normalized template surface. The activations upon the surface were assessed using neuroanatomical labels provided by Destrieux and colleagues^17^.

### Functional connectivity analysis of simulated fMRI signals

**Figure 4:**
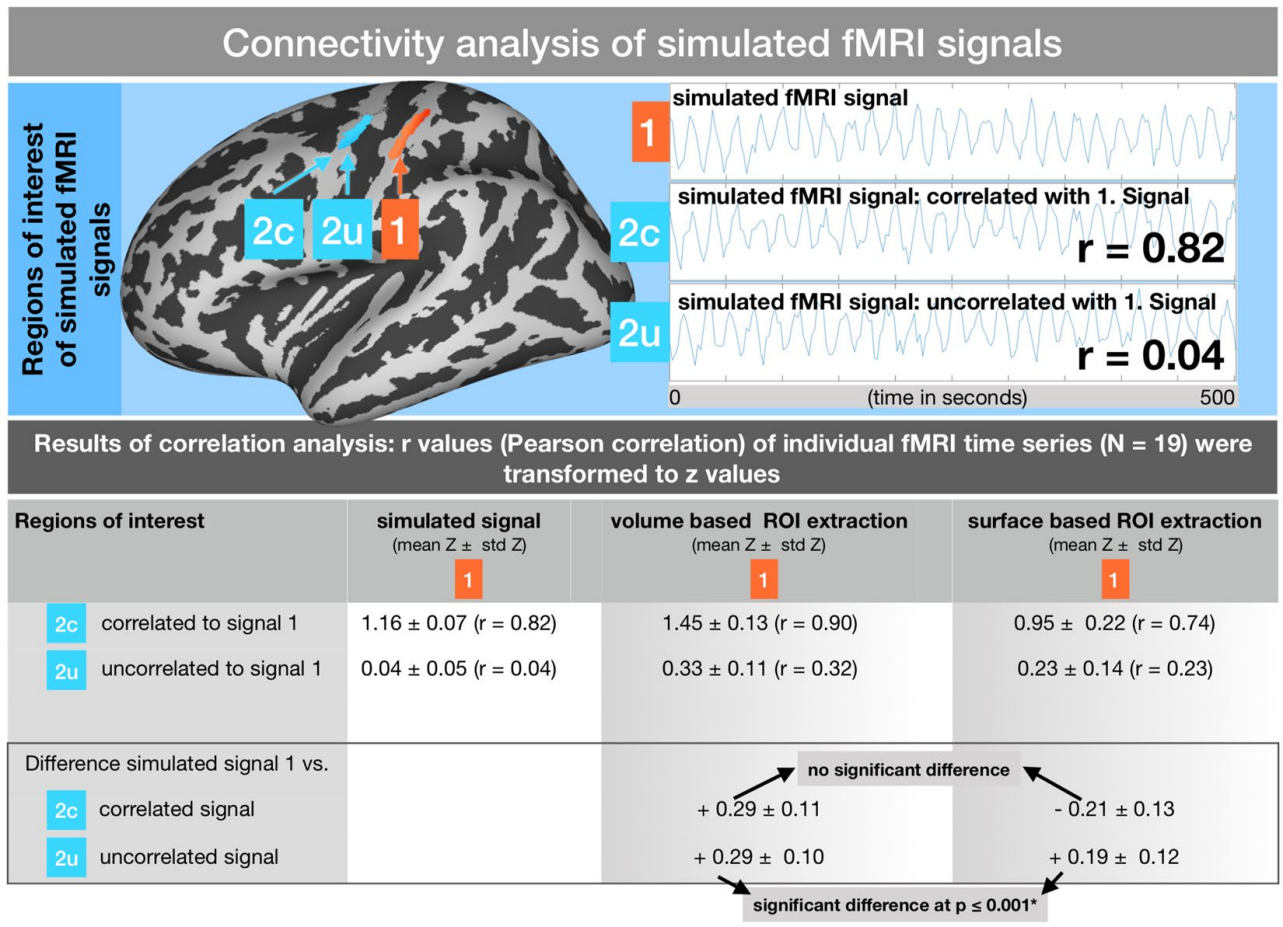
Functional connectivity analysis of simulated fMRI signals within the pre- and postcentral gyri. Upper part: Schematic of the creation of a simulated 0.05 Hz signal (signal 1) in the postcentral region. Correlated (2c) and uncorrelated (2 u) signals placed into the precentral region. Lower part: Results of the functional connectivity analysis between signal 1 and the correlated (2c) as well as the uncorrelated (2 u) signal using the VBA and SBA. The difference between the designed correlation (r = 0.82 for 1~2c and r = 0.04 for 1~2 u) and the actual measured correlation is shown in the lower row. Significant differences between the VBA and SBA are indicated.

To evaluate the effect of a surface-based analysis of functional connectivity, we created artificial fMRI signals (signal 1 in Fig. 4) with a frequency of 0.05 Hz and a random white noise portion of 20%, as well as corresponding signals that were correlated (signal 2c in Fig. 4) and uncorrelated (signal 2 u) to the first signal. For 19 individual rs-fMRI time series (210 images, duration approximately 10 minutes), these signals were created separately and consequently added to regions within the somatosensory (postcentral) and motor (precentral) cortices, as shown in Fig. 4. The amplitude of the simulated signal for each region of interest (ROI) was incrementally increased until the final combined signal (original local fMRI signal and added simulated signal) reached a correlation of at least 95% to the simulated signal. For each individual fMRI time series, the final signal in the postcentral gyrus (1) was correlated between r = 0.80 and r = 0.84 to the correlated signal (2c) and between r = 0.02 and r = 0.06 to the uncorrelated signal (2 u) within the precentral gyrus.

Again, the regions-of-interest (ROIs) within the pre- and postcentral gyrus were created in a normalized DARTEL template, and for each subject, individual ROIs within the individual volume space were made via inverse transformation of the normalized ROI data. After adding the simulated signal to the individual fMRI data in the individual space, we performed functional connectivity analysis between these regions using the following VBA and SBA.

The VBA consisted of the following steps: (1) normalization, (2) smoothing to 9 mm, (3) temporal filtering between 0.01 and 0.1 Hz, (4) removal of the mean cerebrospinal-fluid (CSF) signal by linear regression, and (5) extraction of the ROIs using the defined ROIs in normalized space.

For the SBA, the steps were as follows: (1) temporal filtering between 0.01 and 0.1 Hz and (2) removing the mean CSF signal by linear regression. (3) Eventually, the 3D volumes (210 images) for each subject were mapped to the surface, and normalization (including smoothing to 9 mm within the surface space) was performed upon the surface space. For the surface mapping of the fMRI data, we applied a weighted-mean method that uses a Gaussian kernel for mapping along the normals (weighted from 50% at the boundaries up to 100% at the center); this method is provided by the CAT12 toolbox. (4) Finally, ROI extraction was performed within the surface space.

For connectivity analysis, we performed a pairwise (Pearson) correlation of the mean time-series signals within the postcentral (1) and precentral (2c/2 u) gyrus. For group analysis, a Fisher’s R/Z transformation was performed (z = atanh(r)).

### Statement of human rights

All procedures performed in studies involving human participants were in accordance with the ethical standards of the institutional and/or national research committee (local ethics committee FSU Jena/reference number 4301-01/15) and with the 1964 Helsinki declaration and its later amendments or comparable ethical standards.

### Informed consent

Informed consent was obtained from all individual participants included in the study.

## Results

### GLM analysis of the cortical activation induced by tactile stimulation of the right hand

**Figure 5:**
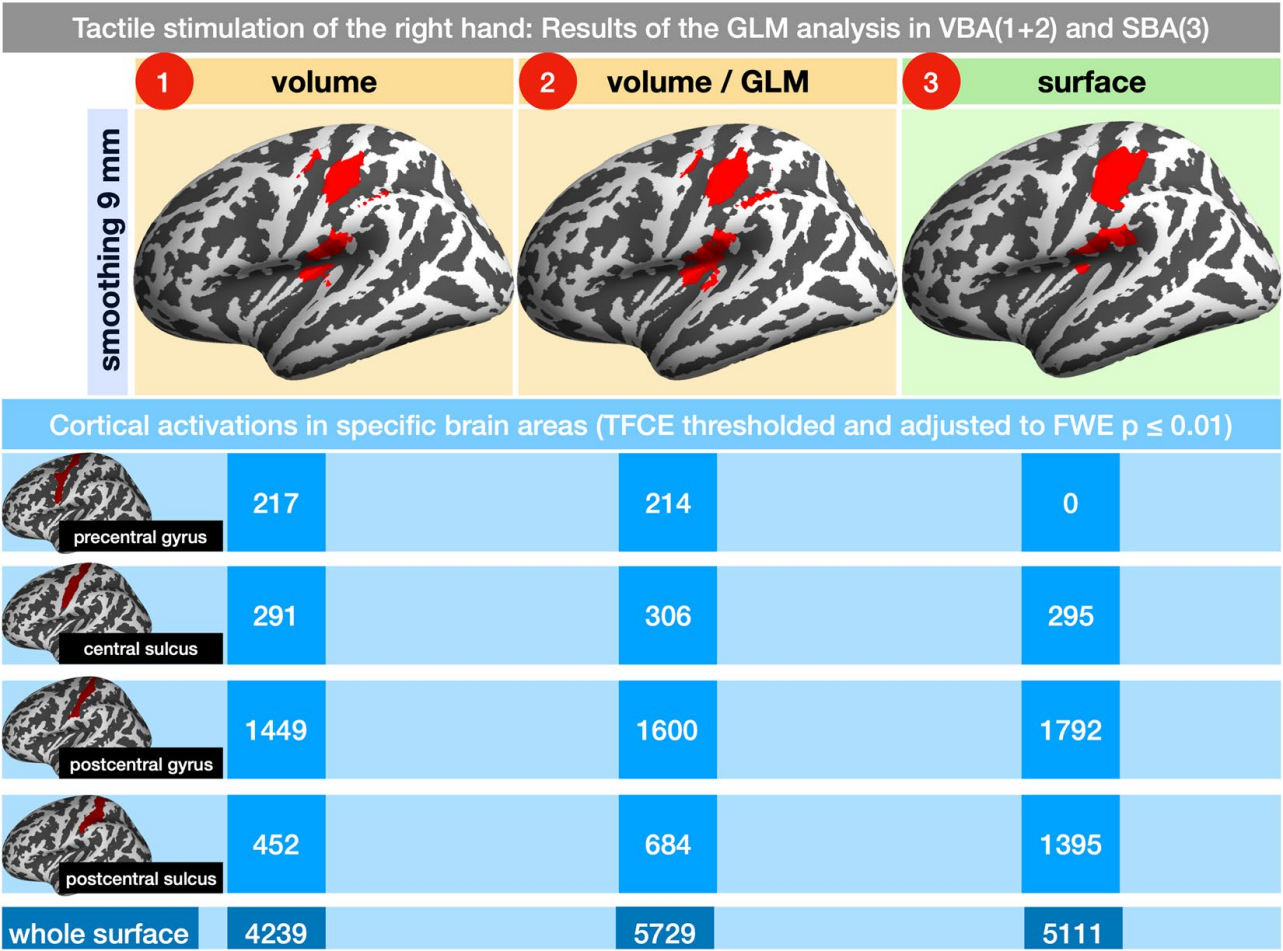
Comparing cortical activation patterns in specific brain regions of volume and SBAs induced by tactile stimulation of the right hand. Cortical activations were counted in 4 brain regions (rows): precentral gyrus, central sulcus, postcentral gyrus and sulcus and results for the 3 different approaches (aligned in columns: 1–2 volume-based, 3 surface-based) were compared. Again, the results of the left cortex are shown (corrected for multiple comparisons using TFCE and adjusted at p ≤ 0.01 FWE).

Both VBA and the SBA returned robust activation patterns within the primary and secondary somatosensory cortices. An overview of all cortical activations in the pre- and postcentral region for the VBA and SBA with different smoothing sizes is provided in Table 1. Figure 5 summarizes a comparison of the activations within the pre- and postcentral cortex between the VBA and the SBA with 9 mm FWHM smoothing. In Fig. S1, a comparison for different smoothing sizes (0–12 mm) is provided.

**Table 1:** SPM activations in brain areas (atlas: Destrieux 2009).

brain localization	atlas description	volume	volume/GLM	surface	volume	volume/GLM	surface	volume	volume/GLM	surface
		*smoothing 6 mm FWHM*			*smoothing 9 mm FWHM*			*smoothing 12 mm FWHM*		
M1	precentral gyrus	24	183	0	217	214	0	326	313	0
central sulcus	central sulcus	208	266	348	291	306	295	519	495	160
S1	postcentral gyrus	1293	1472	1830	1449	1600	1792	1528	1679	1702
postcentral sulcus	postcentral sulcus	655	1130	1564	452	684	1395	452	859	913
parietal association cortex	superior parietal lobule	0	0	0	0	0	0	3	32	0
S2 / operculum	subcentral gyrus and sulcus, insular gyrus, circular and central sulcus of the insula	193	1049	678	1051	1321	730	1136	1516	401
temporal lobule	anterior transverse temporal gyrus	0	93	0	48	229	0	73	297	0

Cortical activations in response to tactile stimulation of fingers 2 and 4 were found in the primary (S1) and secondary (S2) somatosensory cortices, the primary motor (M1) cortex and neighboring anatomical structures such as the central and postcentral sulci, the parietal association cortex and the superior temporal lobule. The results are grouped according to the applied smoothing strength of 6, 9 and 12 mm FWHM. Categories for each data column refer to the applied analysis approach: volume - VBA (normalization and smoothing was performed prior to the estimation of the general linear model [GLM]), volume/GLM - VBA (the GLM was estimated prior to the normalization and smoothing), surface - SBA (normalization and smoothing of the GLM results on the mapped surface).

#### Comparing both VBAs

Performing the GLM analysis prior to the spatial transformation resulted in more significant activations within the primary and secondary somatosensory cortices (S1: 1472 vs. 1293 at 6 mm smoothing, 1600 vs. 1449 at 9 mm smoothing and 1679 vs. 1528 at 12 mm smoothing). However, this was accompanied by an increase in activated brain areas outside the somatosensory cortex. Especially activations within the motor cortex (precentral gyrus: 183 vs. 24 at 6 mm smoothing, 214 vs. 217 at 9 mm smoothing and 313 vs. 326 at 12 mm smoothing), the association cortex (superior parietal lobule: 32 vs. 3 at 12 mm smoothing) and the temporal cortex (superior temporal lobule: 93 vs. 0 at 6 mm smoothing, 229 vs. 48 at 9 mm smoothing and 297 vs. 73 at 12 mm smoothing) were more pronounced.

#### Comparing volume and SBAs

The surface-based GLM analysis revealed cortical activations within the primary and secondary somatosensory cortices (S1: 1830 at 6 mm smoothing, 1792 at 9 mm smoothing, 1702 at 12 mm smoothing; S2: 678 at 6 mm smoothing, 730 at 9 mm smoothing, 401 at 12 mm smoothing).

Brain areas outside the somatosensory cortex, such as the motor cortex (precentral gyrus), the parietal association cortex (superior parietal lobule) and the temporal lobe (anterior transverse temporal gyrus) did not provide any significant activation patterns. However, the structures directly neighboring the primary somatosensory cortex (namely, the central and postcentral sulci) showed very similar activation patterns (central sulcus: 208 (volume) vs. 266 (volume / GLM) vs. 348 (surface) at 6 mm smoothing, 291 vs. 306 vs. 295 at 9 mm smoothing and 519 vs. 495 vs. 160 at 12 mm smoothing; postcentral sulcus: 655 vs. 1130 vs. 1564 at 6 mm smoothing, 452 vs. 684 vs. 1395 at 9 mm smoothing and 452 vs. 859 vs. 913 at 12 mm smoothing)(Fig. 5).

#### Effect of the smoothing strength

As a general trend, we observed that with an increasing smoothing kernel, the amount of activated regions in the VBAs increased, whereas the amount in the SBA decreased (i.e., S1: VBAs 24/183 at 6 mm, 217/214 at 9 mm and 326/313 at 12 mm smoothing, for the SBA: 1830 at 6 mm, 1792 at 9 mm and 1702 at 12 mm smoothing). To test for significance of this observation, we created a GLM where the amount of clusters in the brain areas, described in Table 1, for each approach were the dependent factors and the strength of the kernel (FWHM) and the brain localization were the independent factors. In all 3 approaches the strength of the smoothing filter (6, 9 and 12 mm) was a significant factor to explain the model at p ≤ 0.05.

Figure S1 in the supplementary material shows the effect of the smoothing strength from 0 (no smoothing) up to 12 mm with the VBA and the SBA.

#### Somatotopic finger representation in simulated brain activity

The results of the comparison of the VBA and SBA in GLM analysis with simulated brain activity in the somatotopic regions for fingers D1 and D3 are shown in Fig. S2. The best segregation of individual fingers D1 and D3 was achieved with 3 mm smoothing size in both the VBA and the SBA; the accuracy of the overlap of simulated voxels and activated voxels was 83% in the VBA and 97% in the SBA. With increasing smoothing factor, the activation patterns of D1 and D3 were blurred. In all VBA results, there were false-positive activation patterns within the precentral cortex. Overall, there were more activated voxels in the postcentral region in the VBA (smoothing 0 mm: 16; 3 mm: 27; 6 mm: 81) than in the SBA (0 mm: 11; 3 mm: 23; 6 mm: 44).

### GLM analysis of simulated HRF signals within the primary somatosensory cortex

**Figure 6:**
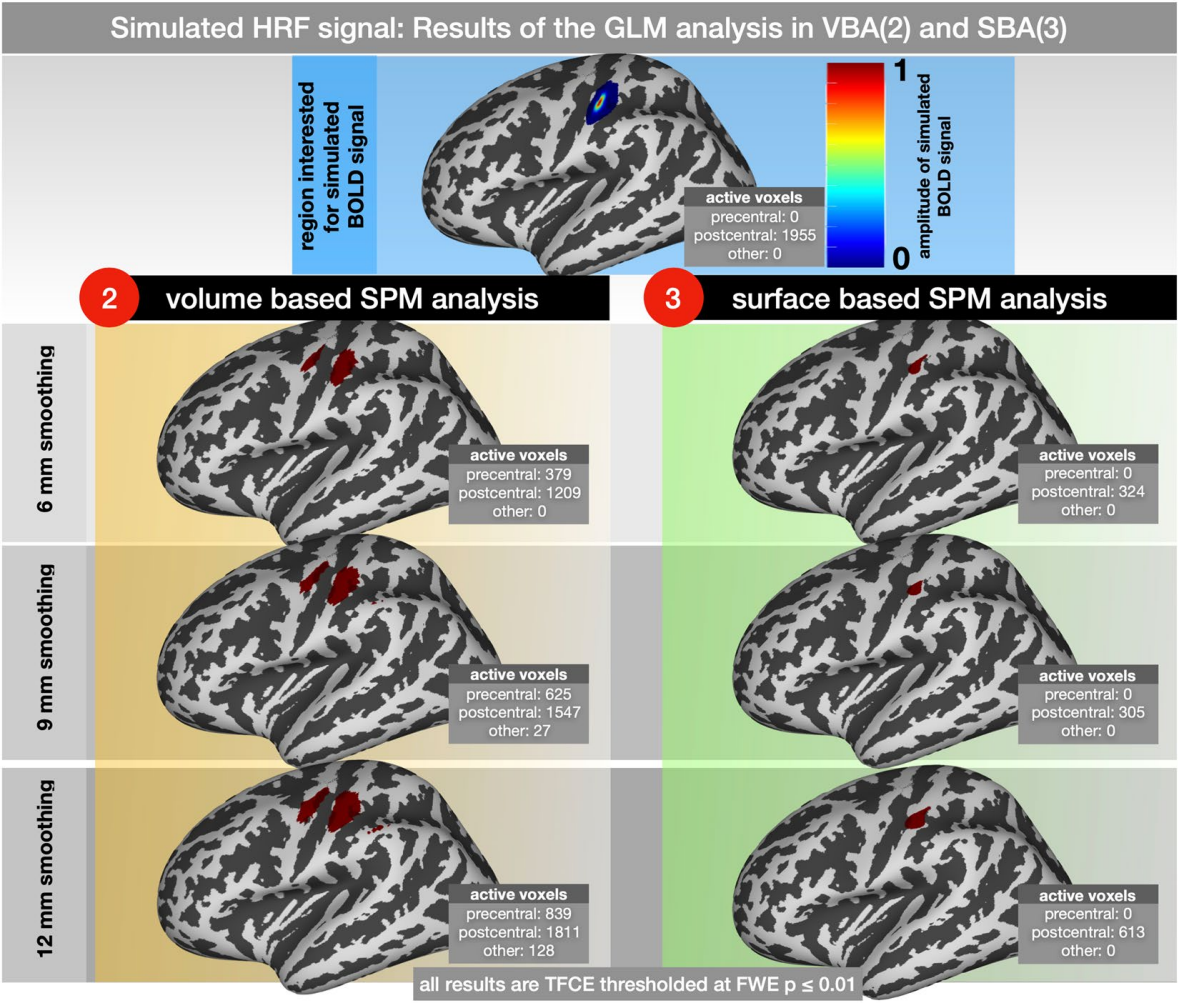
Results of the SPM analysis of a simulated HRF signal. The simulated BOLD signal occurred every 10 s and lasted 1 s. GLM-results were smoothed using 6, 9 and 12 mm; 2nd level results were corrected for multiple comparisons and adjusted at p ≤ 0.05 FWE. The number of active voxels within the precentral and postcentral gyri are displayed for each separate analysis.

Since co-activation within the precentral cortex due to somatosensory stimuli is commonly observed and might be connected to real physiological brain activity, we intended to evaluate the effect of the volume and surface-based GLM analysis on a pre-known cortical signal limited to the postcentral cortex. The results of the volume and surface-based GLM analysis are summarized in Fig. 6.

Volume and SBAs could identify the simulated HRF signal within the primary somatosensory cortex. While using the VBA, the size of the activated brain area increased (1209 at 6 mm, 1547 at 9 mm and 1811 at 12 mm smoothing), and the brain activation pattern identified with the SBA remained more focalized (324 at 6 mm, 305 at 9 mm and 613 at 12 mm smoothing). Using the SBA, no activation patterns were found outside the simulated area in the postcentral cortex. On the contrary, using the VBA, cortical activations were observed in nearby regions in the volume, such as the precentral cortex (379 at 6 mm, 625 at 9 mm and 839 at 12 mm smoothing) and more caudal parietal cortex (0 at 6 mm, 27 at 9 mm and 128 at 12 mm smoothing).

### Connectivity analysis of simulated signals within the primary motor and somatosensory cortices

Connectivity of pairs of correlated (1~2c in Fig. 4) and uncorrelated (1~2 u in Fig. 4) simulated fMRI signals within the pre- and postcentral cortices were analyzed in 19 individual rs-fMRI time series.

#### Functional connectivity of two correlated signals in nearby brain regions

High signal correlations within the pre- and postcentral cortices could be detected using both the volume and surface-based time series extraction approaches. However, while the original correlation between the two signals was designed to have a Pearson correlation coefficient of about r = 0.82, the VBA revealed an increased correlation coefficient of r = 0.90 and the SBA revealed a decreased coefficient of r = 0.74. Within the group analysis, the difference between the volume and SBA did not significantly vary from the original correlation coefficient of r = 0.82 (using a 2-sample t-test on the transformed z values).

#### Functional connectivity in two uncorrelated signals in nearby brain regions

Both methods using surface and volume-based time series extraction showed a considerably increased correlation coefficient for the two uncorrelated signals (simulated signal correlation: r = 0.04, VBA: r = 0.33, SBA: r = 0.23). Comparing the deviation of the average r/z transformed coefficients for each different approach and the original simulated signal, the difference between both approaches was significant in a 2-sample t-test (at p ≤ 0.01). Thus, the deviation of the correlation coefficient was less pronounced with the SBA.

## Discussion

In the present study, we compared the effects of volume-based and surface-based smoothing on activity patterns and functional connectivity of neighboring brain regions. Taking the primary somatosensory and motor cortices as a typical example, we were particularly interested in the effects of signal contamination between these regions.

Tactile stimulation of fingers 2 and 4, as used in the current experiment, produced robust activation patterns in the contralateral primary somatosensory cortex by both the standard volume-based and surface-based smoothing approaches. The spatial location of the maximum activity was in line with previous fMRI studies^18–21^ and showed only slight divergences between volume- and surface-based smoothing.

A main objective of the present study is the comparison of the VBA and SBA regarding brain activity around the central sulcus, namely, the motor (precentral) and somatosensory (postcentral) cortices. By implication, different scientific questions involve different techniques, and this is especially true for fMR imaging^22–24^. We think that our approach and argumentation concerning brain activity using standard 3 T fMR imaging is appropriate for many recent studies investigating the pre- and postcentral regions. However, any kind of applied filter, whether in the spatial or the temporal domain, must be reviewed critically^25,26^. Especially in single-case studies or in special settings such as high-resolution imaging, common spatial filtering can be considered counterproductive^27^. On the other hand, the SBA can potentially improve scenarios where smaller spatial scales (than pre- and central cortex) and functional heterogeneity, i.e., somatotopic, retinotopic or tonotopic representation, are involved^28^. The registration of individual MR data on the surface can improve accuracy and therefore the localization of small cortical structures^29,30^.

### Task-fMRI: GLM

We applied two different pipelines for the VBA (Fig. 2, rows 1 and 2). While the first approach (normalize & smooth > GLM > 2nd-level analysis) resembles the standard SPM pipeline, the second (GLM in native space > normalize & smooth > 2nd-level analysis) is more similar to the FSL pathway^31^. The main purpose of using the second volume approach was to use the same individual 1st-level results for the volume- and surface-based group analyses and therefore make the effects of volume and surface smoothing comparable. The results of the two VBAs slightly differed (Table 1 and Fig. 5) and illustrate the effects of pre- and poststatistics normalization^31^.

The most common approach to compare brain activity using fMR imaging is by comparing time-series data in a voxel-by-voxelwise manner. For group analyses, a spatial normalization to a normalized space has to be performed at some point of the analysis procedure. Volume-based registration includes the whole brain volume, including different tissue types (for a review see Klein^32^). Surface-based methods approach spatial normalization from another perspective. SBAs use surface parameters such as sulci to match corresponding brain regions across individuals (for a review, see Klein^33^). In general, SBAs are based on more accurate coregistration and reduce the brain volume to the cortex itself^34^.

For VBA and SBA, an essential step for the precise anatomical representation of the functional individual data is the precise registration of structural and functional images. Since DARTEL and nonlinear registration increase the accuracy, we applied both methods to register the functional to the structural images^35^.

#### GLM: somatosensory cortex activations

Within the primary somatosensory cortex, we found more activations using surface-based smoothing for each tested kernel size. The extent of the activated brain volume increased with the kernel size of volume-based smoothing, while the activated brain volume stayed relatively stable under different kernel sizes of surface-based smoothing. These findings were in agreement with previous studies that investigated simulated BOLD responses^5^ and experimental fMRI data^36^. The reduced influence of noise outside the gray matter in surface-based smoothing was suggested as a main cause of these differences^37,38^.

Even without spatial smoothing, there was significant brain activation in the somatosensory cortex using the SBA (Fig. S1). With the VBA, no activation survived the correction threshold. This is most likely due to the superior spatial registration and alignment across subjects, especially at the location of the central sulcus^34,39,40^; this matter will be further discussed in the study’s limitations below.

At a smoothing size of 3 mm, the best somatotopic segregation of fingers 1 and 3 was achieved with the VBA and SBA. A similar smoothing size was found to be adequate for finger somatotopy with similar fMRI parameters^41–43^. With the SBA, the overlap of activated and previously stimulated voxels was higher (83% vs 97%) and the overall occurrence of false-positive activations was lower than with the VBA. It is conclusive to assume that the superior registration of the postcentral structures in the SBA is the reason for the better finger segregation in the cortical activation pattern^29^. Although surface mapping of functional MR data has recently been used in other brain regions, such as the visual and auditive^44–46^ systems, to our knowledge, there is no systematic comparison between the VBA and SBA available.

#### GLM: false positive motor cortex activation

The primary research question of the current study was to delineate the effect of volume and surface based smoothing methods on brain areas that are near the activated brain region but not engaged in the experimental task. For our exemplary investigation of the primary motor- and somatosensory cortices, we were interested in the effects of brain responses within the primary somatosensory cortex on the primary motor cortex. Using volume-based smoothing, we found false positive activations within the primary motor cortex for all kernel sizes. In our study, we chose kernel sizes of 6, 9, and 12 mm to cover the most common kernels in recent imaging literature^47^; however, the most common kernel size is the 8-mm FWHM, which is the default value in SPM^39^. The activated part of the primary motor cortex was located in direct (volumetric) proximity to the activated part of the primary somatosensory cortex. From the experimental data alone, one could formally argue that tactile stimulation might also activate the motor cortex. However, this is not supported by previous studies^19,48,49^. More importantly, the similarity of the motor activation pattern between the experimental (Fig. 5) and simulated (Fig. 6) data did not support this argument. Accordingly, no significant activation of the primary motor cortex was found using the surface-based smoothing approach in simulated and experimental data.

The activation of the primary motor cortex can therefore be interpreted as false positive activation caused by an artificial signal contamination across the neighboring sulcal border induced by volume-based smoothing. This interpretation is further strengthened by the finding that the activity in the motor cortex increased with the size of the volume-based smoothing kernel, suggesting a simple blurring effect across the border of the central sulcus.

Although the interpretation of motor activation as artifacts might be straightforward in the current study, such interpretations can be far more difficult in other experimental designs. To stay with the example of the primary somatosensory cortex, one of the most often used stimuli is electrical stimulation of the median nerve due to its simple use, exact controllability in time and strength as well as its associated reliable cortical activations^49–51^. However, the median nerve is a mixed sensory/motor nerve, and it could be argued that electrical activation of this nerve might also provide feedback information that is processed in the primary motor cortex. Because it is currently an unanswered question, any activation of the primary motor cortex due to a median nerve stimulus cannot be definitively differentiated between a blurring effect of the volume-based smoothing and true activation. Similar ambiguities are known for a multitude of spatially adjacent functional systems. Separating cortical responses between neighboring areas using fMRI is particularly important for the interpretation of data obtained by other methods, such as MEG and EEG, which address lower spatial resolution^52–54^. Therefore, surface-based smoothing approaches are important and well suited for answering questions about co-activations of neighboring brain regions.

#### GLM: false negative somatosensory cortex activation in simulated data

We demonstrated that surface-based smoothing can reduce the rate of false-positive activations in the primary motor cortex. Thus, it increases specificity and precision (positive predictive value) in both real and simulated brain activity^37^. At first glance, the opposite is true for the sensitivity of cortical activation within the somatosensory cortex. As shown in Fig. 6, surface-based SPM analysis could identify only 1/6 to 1/3 of the simulated HRF signals in the postcentral region. Instead, the VBA could positively identify more than 90% of the simulated 1955 voxels.

To further investigate this issue, we considered different smoothing sizes (0–12 mm) from the GLM analysis of real brain activity induced by tactile stimulation of fingers 2 and 4 of the right hand (Fig. S1). We observed that the activated brain region within the somatosensory cortex is less significantly influenced by the smoothing size in the SBA than in the VBA. More importantly, the amount of activations in the somatosensory cortex detected by the SBA is slightly above the amount detected by the VBA.

Our results suggest that the loss of sensitivity in the SBA is exclusive to the simulated data. Accordingly, in Fig. S2 (here, we simulated local brain activity of different fingers in the somatosensory cortex), the activated brain regions are less abundant in the SBA. It is plausible to assume that the reason for this discrepancy is the methodological approach here. Simulated HRF is added to resting-state brain activity of each subject in the individual space and is based on an ROI defined in volume space (please see Fig. S3 in the supplementary fur further illustration). Most likely, some SPM activation patterns in the volume are not mapped to the surface, because they are not registered as grey matter. In general this might represent one of the mayor advantages of SBA, because only signals that originate from the cortex are processed in the SBA^55^. This is particularly useful when dealing with artifacts, mislocalizations or superficial vascular artifacts^56,57^.

Nonetheless, we have to acknowledge, that the SBA might trade a reduction of false positive activation patterns with an increase of false negatives as well.

### rs-FMRI: connectivity analysis

Signal contamination between adjacent areas should also affect the analysis of their connectedness. By estimating the functional connectivity between the primary somatosensory and primary motor cortices, we demonstrated increased estimates for the connectedness by using volume-based smoothing. This finding is well in agreement with the blurring effect of the volume-based smoothing kernel that causes a mixture between somatosensory- and motor signals and leading to increased signal correlation. This result is not only of theoretical interest as it might affect results of studies that estimated the functional connectivity between the primary somatosensory and primary motor cortex^58–60^, but it is also generalizable to other studies investigating the functional connectedness of neighboring brain regions. Regarding two signals located in the pre- and postcentral gyrus that were explicitly designed to be uncorrelated, we found inflated functional connectivity in both the VBA and the SBA. Although less pronounced in the SBA, this finding demonstrates that smoothing creates false-positive correlations. Speaking in terms of connectivity, spatial smoothing might fake connectivity where none is present^61,62^. Surface-based smoothing can be considered to improve the validity of these analyses^63–65^.

With respect to the problems induced by spatial smoothing, some studies have renounced smoothing completely for high-field fMRI and specific questions about very restricted brain areas^8,66,67^. However, for most experimental designs, smoothing is a necessary tool as it improves the signal to noise ratio, increases the validity of statistical assumptions underlying the random field theory and decreases statistical effects of inter-individual anatomical differences^2^. Therefore, volume-based smoothing is nevertheless a valuable tool in the analysis of fMRI data. Surface-based smoothing can improve fMRI results compared to volume-based smoothing for certain questions and avoid ambiguity in the interpretation activity and connectivity of spatially neighboring areas. However, surface-based smoothing requires precise surface modeling, which limits its usability. Particularly, surface-based smoothing is only of limited use for subcortical structures and is not applicable for the cerebellar cortex today.

Using the CAT12 toolbox, the SBA described in this study can easily be employed for recent fMRI studies. Thus, the group analysis is performed on a normalized surface, and the standard SPM pathways for the second-level analysis are supported. Furthermore, using the cross-platform file format GIFTI as provided in CAT12, the results can be compared with other systems (i.e., FreeSurfer).

### Study limitations

The main concern of our study was to emphasize the benefits of a SBA in the analysis of fMRI data. However, due to the availability of a vast spectrum of different techniques for both 3D volume- and SBAs, we had to restrict the applied methods to a selection of what we think is a good overview over most recently applied methods^68,69^.

For the anatomical localization and comparisons, we analyzed the activation patterns on the surface with both the 3D volume- and the SBA. Here, we used the Destrieux2009 surface atlas as provided within the CAT12 toolbox^17^. This atlas focuses on sulco-gyral structures, and it is well suited to visualize the effect of volume/surface smoothing upon neighboring gyri in the central region. However, there are many recent atlases that derive from functional network parcellations^8,70^; for instance, the multimodal parcellation of the Human Connectome Project by Glasser and colleagues^71^ is directly available within the CAT12 toolbox.

To compare the results of the volume- and SBAs, we mapped the results of the volume-based analysis onto a standard template surface (32k-mesh HCP compatible) as provided within the CAT12 toolbox. Since we mapped smoothed and group-averaged volume data to the surface, a biased representation has to be taken into account. However, since the target anatomical structures of this study were localized around the central sulcus, which is strongly consistent across subjects, we think that this bias is less impactful^39,40^.

The application of spatial smoothing in neuroimaging has been questioned by many recent studies^40,72^. In general, smoothing can improve the signal-to-noise ratio, compensate for imprecise registration, and help to fulfil statistical conditions. On the downside, spatial smoothing blurs data between tissue compartments and between cortical regions. In addition, smoothing can affect results in task- and rs-fMRI data in single-subject and multisubject experiments^61,62^. Recent approaches circumventing the pitfalls of spatial smoothing include more accurate alignment of the data on the surface^73^, parcellating data^71^, and removing structured noise from the data^74^.

To evaluate the effect of the VBA and the SBA in GLM and connectivity analysis, we used experimental fMRI data and rs-fMRI data with artificial signals to simulate brain activity and connectivity in certain brain regions. The method of using empirical rs-fMRI data and generating simulated images is a common approach for evaluating different strategies of imaging analysis (The Impact of Spatial Normalization Strategies on the Temporal Features of the Resting-State Functional MRI: Spatial Normalization Before rs-fMRI Features Calculation May Reduce the Reliability.). Our main objective was to investigate the effects of the VBA and SBA on brain activity originating from the postcentral cortex. Therefore, defining the ROIs in a volume-based DARTEL template using atlas definitions provided by the CAT12 toolbox is appropriate. However, in different research questions, the ROI acquisition strategy must differ. In addition to different spatial scales (i.e. much larger or much smaller regions), different imaging parameters (i.e. high-resolution fMRI), there are also different surface parcellations to consider^8,22,70,71^.

Furthermore, there is, of course, the problem of multiple comparisons in group comparisons. Here, we applied the method of threshold-free cluster enhancement (TFCE), which can be considered an improvement compared to cluster-based thresholding because it makes the prior definition of a cluster threshold redundant^16^. However, while cluster-based methods can enhance spatial clusters, these methods also appear to be more vulnerable to spatial autocorrelation functions that violate the assumption of a Gaussian distribution in random field theory^75,76^. Since the underlying presumption in the random field theory of uniformly distributed data is not completely adequate in fMRI statistics, nonparametric permutation methods might present an alternative approach^75,77^.

## Conclusion

We demonstrated that surface-based smoothing provides increased spatial acuity of cortical activation compared with volume-based smoothing. The advantages of surface-based smoothing are most pronounced for regions that are close together in the folded areas in the 3D volume because unfolding the brain to the surface space spatially segregates these regions. For these neighboring brain regions, we demonstrated that surface-based smoothing also improved the reliability of analyses of their connectedness, especially by removing artificially increased connectivity of spatial nearby regions. More general benefits are derived from the improved image registration, especially in intersubject analysis.

## Supplementary information

Supplementary Information.

## Data Availability

Anonymized fMRI data and simulated data will be available upon request by the author: stefan.brodoehl@med.uni-med.uni-jena.de.

## References

[1] Puce, A. & Hämäläinen, M. S. A Review of Issues Related to Data Acquisition and Analysis in EEG/MEG Studies. *Brain sciences* **7** (2017). 10.3390/brainsci7060058 PMC5483631 28561761

[2] Friston, K., Ashburner, J., Kiebel, S., Nichols, T. & William, P. *Statistical Parametric Mapping: The Analysis of Functional Brain Images*. (Academic Press, 2007).

[3] Mikl M (2008). Effects of spatial smoothing on fMRI group inferences. Magnetic resonance imaging.

[4] Sacchet MD, Knutson B (2013). Spatial smoothing systematically biases the localization of reward-related brain activity. NeuroImage.

[5] Andrade A (2001). Detection of fMRI activation using cortical surface mapping. Human brain mapping.

[6] Lerch JP, Evans AC (2005). Cortical thickness analysis examined through power analysis and a population simulation. NeuroImage.

[7] Hagler DJ, Saygin AP, Sereno MI (2006). Smoothing and cluster thresholding for cortical surface-based group analysis of fMRI data. NeuroImage.

[8] Glasser MF (2013). The minimal preprocessing pipelines for the Human Connectome Project. NeuroImage.

[9] Fischl B (2012). FreeSurfer. NeuroImage.

[10] Dahnke R, Yotter RA, Gaser C (2013). Cortical thickness and central surface estimation. NeuroImage.

[11] Yotter RA, Dahnke R, Thompson PM, Gaser C (2011). Topological correction of brain surface meshes using spherical harmonics. Human brain mapping.

[12] Gaser C, Volz HP, Kiebel S, Riehemann S, Sauer H (1999). Detecting structural changes in whole brain based on nonlinear deformations-application to schizophrenia research. NeuroImage.

[13] Kiebel SJ, Ashburner J, Poline JB, Friston KJ, MRI PET (1997). coregistration–a cross validation of statistical parametric mapping and automated image registration. NeuroImage.

[14] Erhardt EB, Allen EA, Wei Y, Eichele T, Calhoun VD (2012). SimTB, a simulation toolbox for fMRI data under a model of spatiotemporal separability. NeuroImage.

[15] Martuzzi R, van der Zwaag W, Farthouat J, Gruetter R, Blanke O (2014). Human finger somatotopy in areas 3b, 1, and 2: a 7T fMRI study using a natural stimulus. Human brain mapping.

[16] Smith SM, Nichols TE (2009). Threshold-free cluster enhancement: addressing problems of smoothing, threshold dependence and localisation in cluster inference. NeuroImage.

[17] Destrieux C, Fischl B, Dale A, Halgren E (2010). Automatic parcellation of human cortical gyri and sulci using standard anatomical nomenclature. NeuroImage.

[18] Ann Stringer E (2014). Distinct fine-scale fMRI activation patterns of contra- and ipsilateral somatosensory areas 3b and 1 in humans. Human brain mapping.

[19] Klingner CM (2011). Functional deactivations: multiple ipsilateral brain areas engaged in the processing of somatosensory information. Human brain mapping.

[20] Ruben J (2006). Sub-area-specific Suppressive Interaction in the BOLD responses to simultaneous finger stimulation in human primary somatosensory cortex: evidence for increasing rostral-to-caudal convergence. Cerebral cortex (New York, N.Y.: 1991).

[21] Tal Z, Geva R, Amedi A (2017). Positive and Negative Somatotopic BOLD Responses in Contralateral Versus Ipsilateral Penfield Homunculus. Cerebral cortex (New York, N.Y.: 1991).

[22] Eickhoff, S. B., Yeo, B. T. T. & Genon, S. Imaging-based parcellations of the human brain. *Nature reviews. Neuroscience* **19**, 672–686. 10.1038/s41583-018-0071-7 30305712

[23] Poldrack, R. A. & Farah, M. J. Progress and challenges in probing the human brain. *Nature* **526**, 371–379. 10.1038/nature15692 26469048

[24] Soares JM (2016). A Hitchhiker’s Guide to Functional Magnetic Resonance Imaging. Frontiers in neuroscience.

[25] Friston, K. J. *et al*. To smooth or not to smooth? Bias and efficiency in fMRI time-series analysis. *NeuroImage* **12**, 196–208. 10.1006/nimg.2000.0609 10913325

[26] Caballero-Gaudes C, Reynolds RC (2017). Methods for cleaning the BOLD fMRI signal. NeuroImage.

[27] Khan, R. *et al*. Surface-based analysis methods for high-resolution functional magnetic resonance imaging. *Graphical models* **73**, 313–322. 10.1016/j.gmod.2010.11.002 PMC3223917 22125419

[28] Van Essen DC (2004). Surface-based approaches to spatial localization and registration in primate cerebral cortex. NeuroImage.

[29] Pfannmöller, J. P., Greiner, M., Balasubramanian, M. & Lotze, M. High-resolution fMRI investigations of the fingertip somatotopy and variability in BA3b and BA1 of the primary somatosensory cortex. *Neuroscience* **339**, 667–677. 10.1016/j.neuroscience.2016.10.036 27777015

[30] Henriksson L, Karvonen J, Salminen-Vaparanta N, Railo H, Vanni S (2012). Retinotopic maps, spatial tuning, and locations of human visual areas in surface coordinates characterized with multifocal and blocked FMRI designs. PloS one.

[31] Poldrack, R. A., Nichols, T. & Mumford, J. *Handbook of Functional MRI Data Analysis*. (Cambridge University Press, 2011).

[32] Klein A (2009). Evaluation of 14 nonlinear deformation algorithms applied to human brain MRI registration. NeuroImage.

[33] Klein A (2010). Evaluation of volume-based and surface-based brain image registration methods. NeuroImage.

[34] Tucholka A, Fritsch V, Poline J-B, Thirion B (2012). An empirical comparison of surface-based and volume-based group studies in neuroimaging. NeuroImage.

[35] Pizzagalli F, Auzias G, Delon-Martin C, Dojat M (2013). Local landmark alignment for high-resolution fMRI group studies: toward a fine cortical investigation of hand movements in human. Journal of neuroscience methods.

[36] Anticevic A (2008). Comparing surface-based and volume-based analyses of functional neuroimaging data in patients with schizophrenia. NeuroImage.

[37] Jo HJ (2007). Spatial accuracy of fMRI activation influenced by volume- and surface-based spatial smoothing techniques. NeuroImage.

[38] Kiebel SJ, Goebel R, Friston KJ (2000). Anatomically informed basis functions. NeuroImage.

[39] Coalson, T. S., Essen, D. C. V. & Glasser, M. F. Lost in Space: The Impact of Traditional Neuroimaging Methods on the Spatial Localization of Cortical Areas. *bioRxiv* (2018). 10.1073/pnas.1801582115 PMC6142239 29925602

[40] Glasser MF (2016). The Human Connectome Project’s neuroimaging approach. Nature neuroscience.

[41] van Westen, D. *et al*. Fingersomatotopy in area 3b: an fMRI-study. *BMC neuroscience* **5**, 28. 10.1186/1471-2202-5-28 PMC517711 15320953

[42] Nelson, A. J. & Chen, R. Digit somatotopy within cortical areas of the postcentral gyrus in humans. *Cerebral cortex* **18**, 2341–2351 *(New York, N.Y.: 1991)*. 10.1093/cercor/bhm257 18245039

[43] Schweizer, R., Voit, D. & Frahm, J. Finger representations in human primary somatosensory cortex as revealed by high-resolution functional MRI of tactile stimulation. *NeuroImage* **42**, 28–35. 10.1016/j.neuroimage.2008.04.184 18550386

[44] Arcaro, M. J., McMains, S. A., Singer, B. D. & Kastner, S. Retinotopic organization of human ventral visual cortex. *The Journal of neuroscience: the official journal of the Society for Neuroscience* **29**, 10638–10652. 10.1523/JNEUROSCI.2807-09.2009 PMC2775458 19710316

[45] Humphries, C., Liebenthal, E. & Binder, J. R. Tonotopic organization of human auditory cortex. *NeuroImage* **50**, 1202–1211. 10.1016/j.neuroimage.2010.01.046 PMC2830355 20096790

[46] Ahveninen, J. *et al*. Intracortical depth analyses of frequency-sensitive regions of human auditory cortex using 7TfMRI. *NeuroImage* **143**, 116–127. 10.1016/j.neuroimage.2016.09.010 PMC5124525 27608603

[47] Carp J (2012). The secret lives of experiments: methods reporting in the fMRI literature. NeuroImage.

[48] Hlushchuk Y, Hari R (2006). Transient suppression of ipsilateral primary somatosensory cortex during tactile finger stimulation. The Journal of neuroscience: the official journal of the Society for Neuroscience.

[49] Nihashi T (2005). Contralateral and ipsilateral responses in primary somatosensory cortex following electrical median nerve stimulation–an fMRI study. Clinical neurophysiology: official journal of the International Federation of Clinical Neurophysiology.

[50] Backes WH, Mess WH, van Kranen-Mastenbroek V, Reulen JP (2000). Somatosensory cortex responses to median nerve stimulation: fMRI effects of current amplitude and selective attention. Clinical neurophysiology: official journal of the International Federation of Clinical Neurophysiology.

[51] Klingner CM, Hasler C, Brodoehl S, Witte OW (2010). Dependence of the negative BOLD response on somatosensory stimulus intensity. NeuroImage.

[52] Karhu J, Tesche CD (1999). Simultaneous early processing of sensory input in human primary (SI) and secondary (SII) somatosensory cortices. Journal of neurophysiology.

[53] Klingner CM (2015). Parallel processing of somatosensory information: Evidence from dynamic causal modeling of MEG data. NeuroImage.

[54] Mideksa KG (2012). Source analysis of median nerve stimulated somatosensory evoked potentials and fields using simultaneously measured EEG and MEG signals. Conference proceedings:… Annual International Conference of the IEEE Engineering in Medicine and Biology Society. IEEE Engineering in Medicine and Biology Society. Annual Conference.

[55] Jo, H. J. *et al*. Surface-based functional magnetic resonance imaging analysis of partial brain echo planar imaging data at 1.5 T. *Magnetic resonance imaging* **27**, 691–700. 10.1016/j.mri.2008.09.002 19036544

[56] Ress, D., Glover, G. H., Liu, J. & Wandell, B. Laminar profiles of functional activity in the human brain. *NeuroImage* **34**, 74–84. 10.1016/j.neuroimage.2006.08.020 17011213

[57] Moon, C.-H., Fukuda, M., Park, S.-H. & Kim, S.-G. Neural interpretation of blood oxygenation level-dependent fMRI maps at submillimeter columnar resolution. *The Journal of neuroscience: the official journal of the Society for Neuroscience* **27**, 6892–6902. 10.1523/JNEUROSCI.0445-07.2007 PMC6672231 17596437

[58] Fang X (2016). Disrupted effective connectivity of the sensorimotor network in amyotrophic lateral sclerosis. Journal of neurology.

[59] McGregor HR, Gribble PL (2017). Functional connectivity between somatosensory and motor brain areas predicts individual differences in motor learning by observing. Journal of neurophysiology.

[60] Zhou FQ (2015). Intrinsic functional plasticity of the sensory-motor network in patients with cervical spondylotic myelopathy. Scientific reports.

[61] Liu P, Calhoun V, Chen Z (2017). Functional overestimation due to spatial smoothing of fMRI data. Journal of neuroscience methods.

[62] Chen Z, Calhoun V (2018). Effect of Spatial Smoothing on Task fMRI ICA and Functional Connectivity. Frontiers in neuroscience.

[63] Zuo, X.-N. *et al*. Toward reliable characterization of functional homogeneity in the human brain: preprocessing, scan duration, imaging resolution and computational space. *NeuroImage* **65**, 374–386. 10.1016/j.neuroimage.2012.10.017 PMC3609711 23085497

[64] Yeo, B. T. T. *et al*. The organization of the human cerebral cortex estimated by intrinsic functional connectivity. *Journal of neurophysiology* **106**, 1125–1165. 10.1152/jn.00338.2011 PMC3174820 21653723

[65] Seibert, T. M. & Brewer, J. B. Default network correlations analyzed on native surfaces. *Journal of neuroscience methods* **198**, 301–311. 10.1016/j.jneumeth.2011.04.010 PMC3111080 21514321

[66] Sladky R (2018). Unsmoothed functional MRI of the human amygdala and bed nucleus of the stria terminalis during processing of emotional faces. NeuroImage.

[67] Gazzola V, Keysers C (2009). The observation and execution of actions share motor and somatosensory voxels in all tested subjects: single-subject analyses of unsmoothed fMRI data. Cerebral cortex (New York, N.Y.: 1991).

[68] Morgan, V. L., Dawant, B. M., Li, Y. & Pickens, D. R. Comparison of fMRI statistical software packages and strategies for analysis of images containing random and stimulus-correlated motion. 10.1016/j.compmedimag.2007.04.002 PMC2570159 17574816

[69] Calhoun, V. D. *et al*. The impact of T1 versus EPI spatial normalization templates for fMRI data analyses. 10.1002/hbm.23737 PMC5565844 28745021

[70] Gordon, E. M. *et al*. Generation and Evaluation of a Cortical Area Parcellation from Resting-State Correlations. *Cerebral cortex* **26**, 288–303 *(New York, N.Y.: 1991)*. 10.1093/cercor/bhu239 PMC4677978 25316338

[71] Glasser MF (2016). A multi-modal parcellation of human cerebral cortex. Nature.

[72] Turner, R. & Geyer, S. Comparing like with like: the power of knowing where you are. *Brain connectivity* **4**, 547–557. 10.1089/brain.2014.0261 PMC4146387 24999746

[73] Robinson EC (2018). Multimodal surface matching with higher-order smoothness constraints. NeuroImage.

[74] Glasser, M. F. *et al*. Using temporal ICA to selectively remove global noise while preserving global signal in functional MRI data. **undefined**. 10.1016/j.neuroimage.2018.04.076 PMC6237431 29753843

[75] Eklund A, Nichols TE, Knutsson H (2016). Cluster failure: Why fMRI inferences for spatial extent have inflated false-positive rates. Proceedings of the National Academy of Sciences of the United States of America.

[76] Bansal R, Peterson BS (2018). Cluster-level statistical inference in fMRI datasets: The unexpected behavior of random fields in high dimensions. Magnetic resonance imaging.

[77] Hayasaka, S. & Nichols, T. E. Validating cluster size inference: random field and permutation methods. *NeuroImage* **20**, 2343–2356. 10.1016/j.neuroimage.2003.08.003 14683734

