# Supplementary material for: Surface-based analysis increases the specificity of cortical activation patterns and connectivity results
Source: Sci Rep. 2020 Mar 31;10:5737. doi: 10.1038/s41598-020-62832-z (PMC7109138; doi:10.1038/s41598-020-62832-z)

Surface-based analysis increases the specificity of cortical activation patterns and connectivity results

Stefan Brodoehl; Christian Gaser; Robert Dahnke; Otto W. Witte; Carsten M. Klingner

Supplementary

Figure S1: Effect of smoothing strength in GLM analysis 1

Figure S2: GLM analysis of simulated HRF signals within the cortical representation of individual fingers (D1 + D3) in BA3b 1

Figure S3: Illustration of mapping a simulated ROI to the surface 2

Figure S1: Effect of smoothing strength in GLM analysis

Effect of different smoothing sizes (no smoothing (0 mm) up to 12 mm) on the results of the GLM analysis comparing volume- (2) and surface- (3) based approaches. The subjects (N = 19) were stimulated at fingers 2 and 4 of the right hand. SPM activation maps of the contralateral left cortex are shown. For better comparison, the results of the volume-based GLM were mapped onto the standard template surface (32k mesh HCP compatible) as provided in the CAT12 toolbox. The results were corrected for multiple comparisons using TFCE and adjusted at $p \leq 0.01$ FWE.

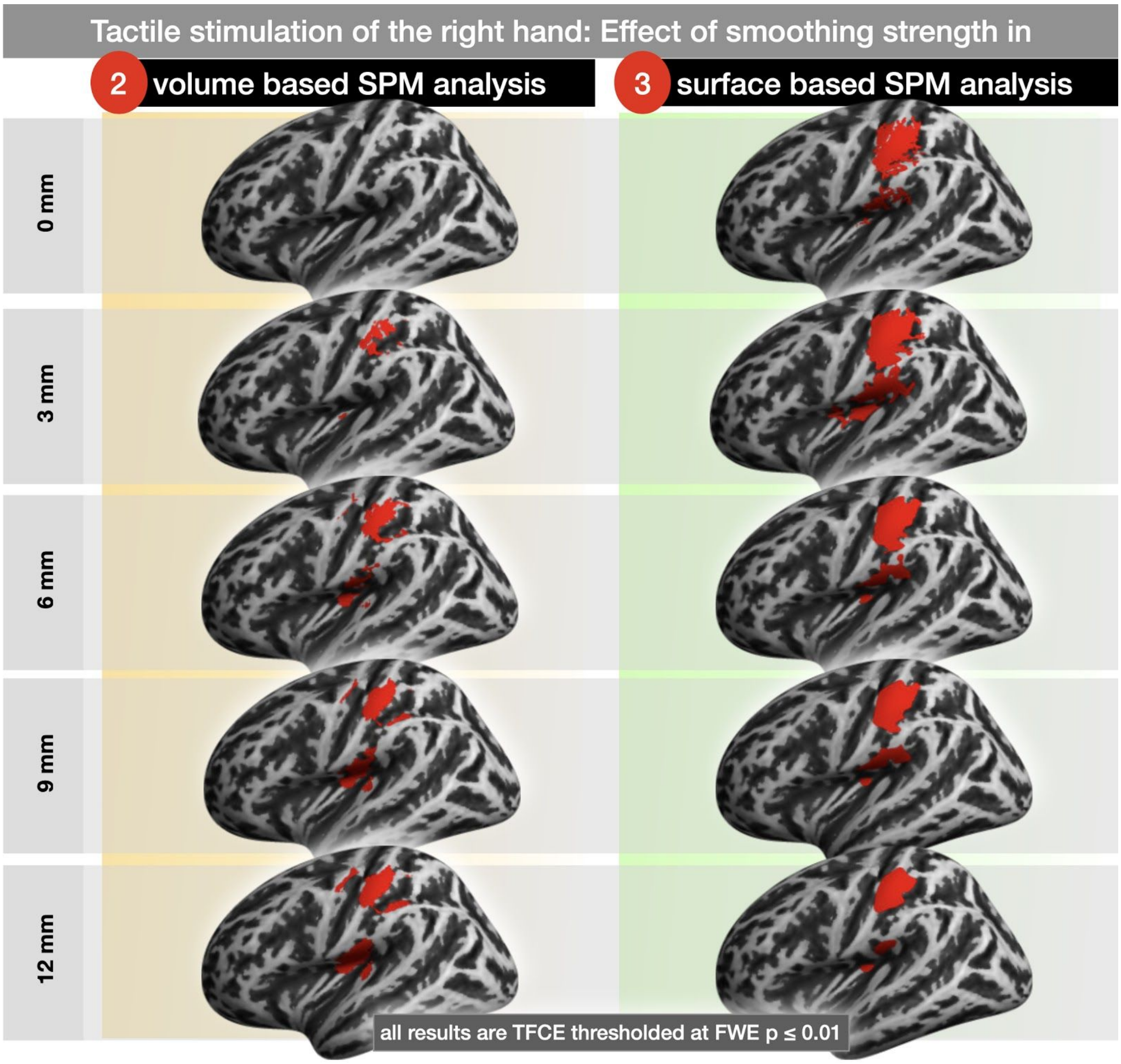

Figure S2: GLM analysis of simulated HRF signals within the cortical representation of individual fingers (D1 + D3) in BA3b

The effect of different smoothing sizes (no smoothing up to 6 mm) upon simulated HRF signals within the somatotopic representation of fingers D1 and D3 within Brodmann area 3b differ in the volume- (2) and surface (3)-based approaches. In both approaches, a smoothing size of 3 mm was appropriate to differentiate between the cortical activation pattern of fingers D1 and D3, while at smoothing sizes above and below, no differentiation was achieved. Furthermore, analogous to the previous results, volume-based smoothing was associated with false-positive activations in the precentral gyrus in BA4. The 2nd level results (N = 19) were corrected for multiple comparisons and adjusted at $p \leq 0.05$ FWE.

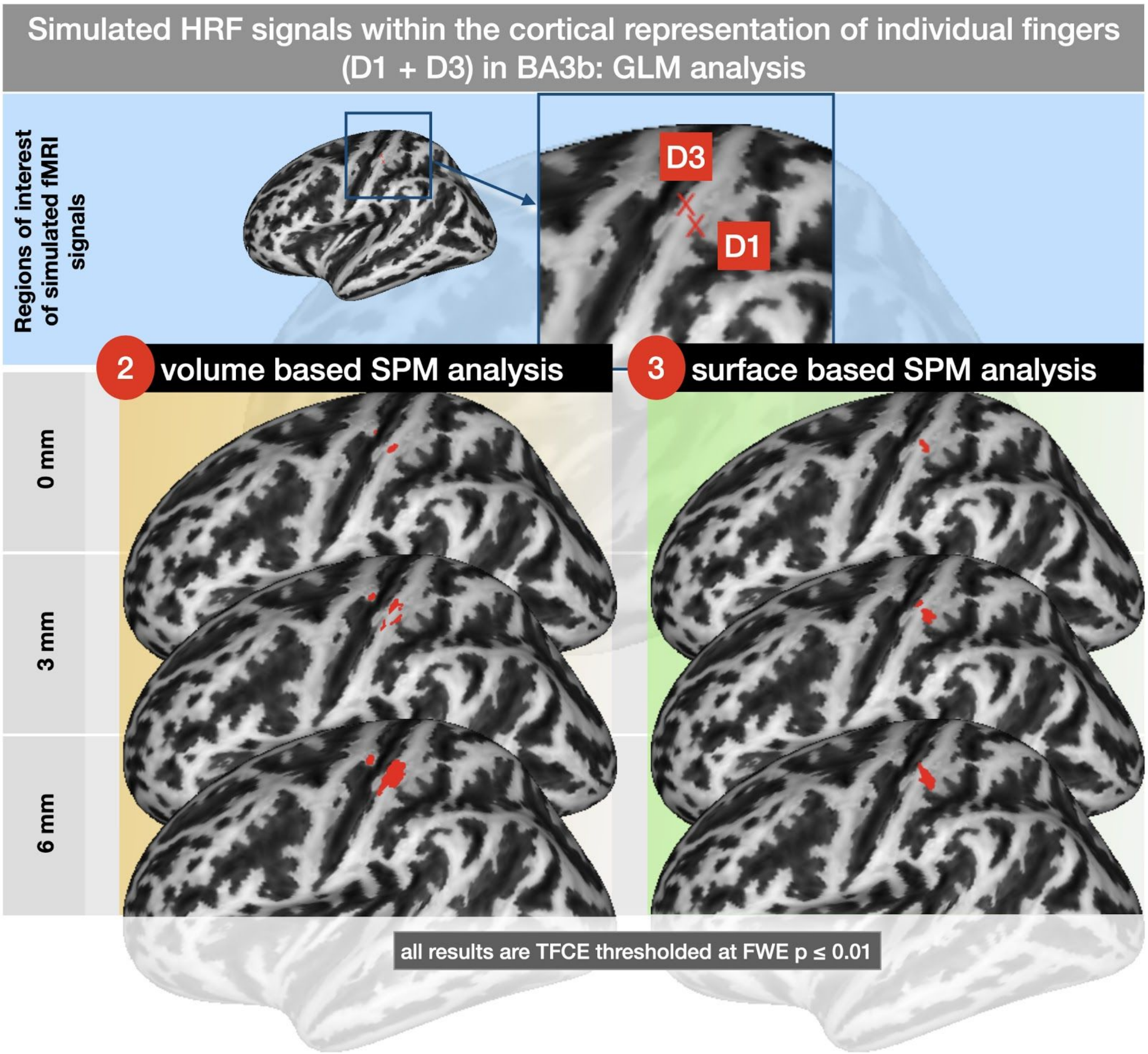

Figure S3: Illustration of mapping a simulated ROI to the surface

Using a high-resolution T1 image a ROI (ø 3mm) with a defined intensity of 1 at the center and decreasing intensity at the periphery (using a weighted Gaussian field) in the volume domain (upper row). By using the CAT12 toolbox the ROI is mapped to the surface (lower row).

While the ROI definition in the volume includes grey- and white matter tissue, the surface domain is planar. Therefore, the tissue, that is not registered as grey matter is not mapped to the surface.

(Please note, this figure is for illustration only. The precise approach to define the ROIs in individual volumes is described in section 2.4.3)

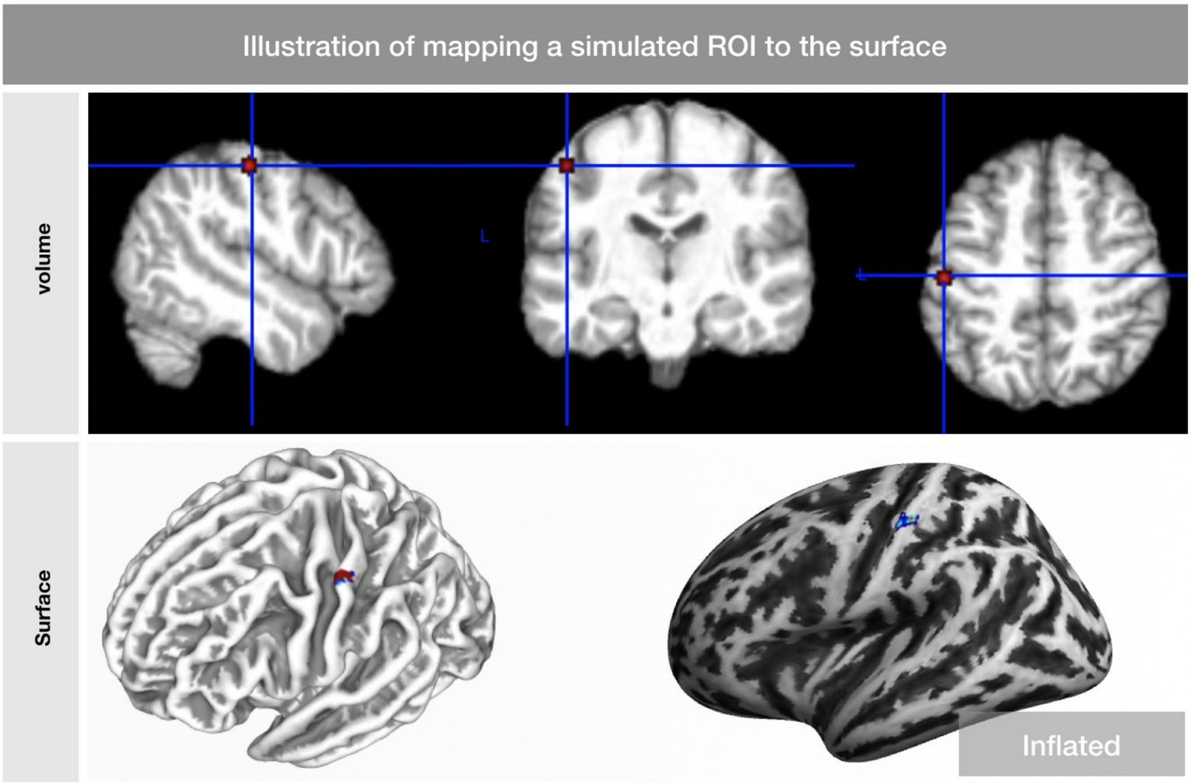

Supplement: Supplementary file 1 — Supplementary Information. [file 41598_2020_62832_MOESM1_ESM.pdf]
